# Parasitological Examination of the Digestive System of Wild Boar from a Practical Point of View—Endoparasitological Sampling under Field Conditions

**DOI:** 10.3390/mps7040065

**Published:** 2024-08-21

**Authors:** Csaba Farkas, Alexandra Juhász, Balázs Fekete, Borisz Egri

**Affiliations:** 1 Department of Animal Science, Albert Kázmér Faculty of Mosonmagyaróvár, Wittmann Antal Multidisciplinary Doctoral School, Széchenyi István University, H-9200 Mosonmagyaróvár, Hungary; egri.borisz@ga.sze.hu; 2 Tropical Disease Biology, Liverpool School of Tropical Medicine, Liverpool L3 5QA, UK; alexandra.juhasz@lstmed.ac.uk; 3 Institute of Medical Microbiology, Semmelweis University, H-1089 Budapest, Hungary; 4 Nexum Veterinary Medicine and Service Ltd., H-2440 Százhalombatta, Hungary; drbubuhu@gmail.com

**Keywords:** dissection, helminths of digestive systems, methodology, sample collection, wild boar

## Abstract

From 2015 to 2023, we conducted a comprehensive study in the 11,893-hectare hunting area managed by the Marcal-Bitvaközi Hunting Company, characterised by its substantial wild boar population. The research was carried out across various settings, including a free-range wild boar garden during large-scale hunts and free-living areas during individual hunts. We examined 216 wild boars in total, with 173 individuals from free-living areas and 43 from free-range areas. Throughout the sample collection process, we encountered numerous technical challenges that are infrequently detailed in the professional literature, often mentioned only tangentially. This oversight in existing publications neglects the significance of addressing field sampling difficulties, which are crucial for ensuring the precision and accuracy of research. This paper details the equipment requirements, sampling methodologies, and practical solutions to streamline fieldwork. While our primary focus was on endoparasitic infections of the stomach and small intestine, the described methodologies and findings are broadly applicable to research involving all internal organs.

## 1. Introduction

The wild boar (*Sus scrofa* Linnaeus, 1758) The wild boar (*Sus scrofa* Linnaeus, 1758) is a critical species in European and Hungarian game management. Despite the significant impact of endoparasites on the health and condition of wild boars, the study of parasites, particularly those in the small intestine, has been largely overlooked. Effective wildlife management necessitates a balance between profit-driven strategies and the ecological equilibrium of wildlife and their habitats [1]. Maintaining the health of the wild boar population is integral to this balance, given their economic importance and the zoonotic risks posed by the parasites they harbour [2]. The zoonotic potential of these parasites is heightened by increasing human-wildlife interactions due to urbanisation, such as people residing in small villages within hunting areas and activities like farming, hiking, and forest traversal. Additionally, the health of individuals involved in wildlife management must be safeguarded [3]. Consequently, it is crucial to consider these risks during sample collection.

In the endoparasitological examination of wild boars, especially when processing a large number of samples, the methods, tools, and techniques employed are critical. Each dissection poses a significant physical burden on the researcher and examiner. To facilitate sample collection during large-scale hunts, where other tasks may also demand attention, it is essential to streamline and simplify the collection process.

Specialists in this field typically conduct research by collecting stool samples and processing them using various laboratory methods [4]. Among the qualitative faecal examination techniques, sedimentation enrichment [5] and the flotation technique [6] are most commonly used, often in combination [7,8]. For more accurate results, some researchers combine quantitative and qualitative coprological methods [9,10]. Researchers frequently utilise multiple standard parasitological coprodiagnostic methods to detect eggs in faeces [11,12].

While faecal examinations effectively determine if an animal is infected and identify the parasite species, this method has limitations. In herd-living animals, it is challenging to attribute a faecal sample to a specific individual. This method does not provide information about the sex, age, health, or condition of the animal from which the sample originated. Additionally, it is difficult to ascertain the number of samples collected from the same animal, which limits the ability to infer infection rates at the herd level. Despite these limitations, faecal examinations are well-suited for identifying parasite species and the extent of worm infections in animals actively shedding eggs.

Dissection-based parasitological sample collection is the most reliable method for accurately assessing population-level infection rates [4,13,14,15,16]. This approach allows for the collection of adult and juvenile parasites from various body parts and organs for further examination and identification. Dissections also enable the determination of the infected individual’s age and sex, as well as the impact of the infection on the animal’s condition and life. Observing the animal’s behaviour before dissection can provide additional insights into disease symptoms.

Previous research of a similar nature (Table 1) was often conducted under calmer conditions, with less physical strain. These studies typically involved smaller herds [17,18,19], and samples were sometimes taken from partially processed wild animals or from individuals in breeding programs.

When collecting our research samples, we conducted our experiments under challenging conditions, similar to the approach used by Varga (2006), who investigated parasite infestations in wild boars during social hunts in free-range and free-living areas from 1996 to 2004 [24]. It is often necessary to simplify and expedite the processes while maintaining the accuracy of the administration and the integrity of the samples, ensuring their subsequent processing is unaffected.

Our methodology aims to facilitate and expedite the collection of endoparasitological samples from wild boars with maximum precision, cleanliness, and accuracy. We provide a comprehensive description of the entire sampling process, highlighting refinements and simplifications in dissection techniques identified during our research. We differentiate between conditions of individual hunting, involving a small number of samples (1–2 individuals), and social hunting, where a large number of samples (between 5 and 50 individuals) must be collected in a short period. Additionally, we describe the tools appropriate for each scenario.

## 2. Design of the Foundation of Our Experiences

Our recommendations were founded on facts based on our experiences [23].

### 2.1. Location of Sample Collection

Our study was conducted from 2015 to 2023 in the Marcal-Bitvaközi Hunting Company area, located in Central Europe, within the Carpathian Basin, specifically in the Somló landscape of Hungary. The area comprises two large forest blocks, natural streams, and agricultural fields. The primary objective of our research was to compare infections of *Ascaris suum* (Goeze, 1782) (Ascaridida, Ascarididae) and *Macracanthorhynchus hirudinaceus* (Pallas, 1781) (Acanthocephala, Oligacanthorhynchidae) in free-living and free-range wild boar herds. We examined 173 individuals from free-living areas and 43 from free-range garden hunts. Among the 173 wild boars from free-living areas, 82 were female, and 91 were male. In the free-range garden, 22 were female, and 21 were male. The sample included 30 juveniles, 107 subadults, and 79 adult wild boars [23].

During the initial examination of these 216 wild boars, we encountered numerous challenges that, while seemingly minor, significantly impacted accuracy and time efficiency. To address these issues, we implemented techniques designed to eliminate such difficulties, ensuring more precise and efficient sample collection and analysis.

### 2.2. Materials and Equipment

#### 2.2.1. Equipment for Collecting Samples on Individual Hunts

The essential equipment background of our sample collection activities in this way is as follows:

- Large foil bag (capacity of 200 L or more) or construction industry covering foil;
- Powerful (20,000 lumens), long-lasting (minimum 2–3 h) headlamp;
- Particularly sharp knife, medical scalpel;
- Sharpening steel;
- Bone saw or splitting axe;
- Several pairs of medical or thicker rubber gloves;
- Medical mask;
- Container suitable for transporting the sample and storing it until processing, or a plastic bag (thick-walled, extra strong construction industry foil bag);
- Strong, 3–4 mm thick binding twine;
- Alcoholic marker pen;
- White, writable adhesive tape;
- Pen;
- Covered writing board;
- Pre-printed sample collection data sheet, which contains the behaviour, gender, estimated age, visceral weight, condition, and health status of the wild boar that can be detected and assessed by visual inspection before the shoot.

#### 2.2.2. Equipment for Collecting Samples on Large-Scale Hunting

In addition to the above, the following is also needed:

- Instead of a foil bag, a collection container with a capacity of 15–20 L that can be closed with a lid;
- White insulating tape

#### 2.2.3. Equipment Required for Sample Selection

- Forceps;
- Glass sheet (approx. 80 cm × 120 cm);
- Strainer;
- Lighting (battery or fixed power supply);
- A small or medium-sized, preferably dark-coloured bowl;
- Rubber glass cleaning paddle.

## 3. Detailed Procedure

### 3.1. The First Phase of Sample Collection

#### 3.1.1. Collecting Samples on Individual Hunts

Our examination process begins with the detection and observation of the game before the shoot [25]. Indicators such as behaviour, movement, coughing, strength, social interaction habits, and body size or morphology can suggest illness. In wildlife management and research, it is crucial to target and eliminate any individual displaying signs of illness. Rarely do we have the opportunity to examine live wild animals and correlate findings with their parasitological burden. The parasitic infections of Hungarian wild animals are often poorly understood, even for the most common infections [26]. Distinguishing infected animals from healthy ones is challenging, as those appearing healthy and vigorous can be heavily infected. Animals that die from parasitic infections are seldom examined for this reason, as deaths are usually attributed to other causes, and samples often degrade naturally without human intervention [26].

After shooting, the game should be retrieved immediately, and internal organs should be removed promptly. Ideally, dissection occurs on the same day, with special attention to parasites in the muscles. The carcass is thoroughly examined, starting with an incision from the ventral side. For males, the penis and testicles are removed, followed by opening the abdominal wall from the sternum to the groin [25]. With the animal on its back, incisions are made towards the pelvis with the blade edge facing outward. The anus is tied off with 15–20 cm of twine to prevent contamination and sample loss, then pulled into the abdominal cavity.

Using a bone saw or splitting axe, the pelvic bone and sternum are cut to expose the chest cavity. An incision is made on both sides of the oesophagus and larynx up to the tongue base; then, these structures are pulled into the chest cavity. The heart, lungs, and connective tissues are cut, along with the diaphragm, up to one-third along the ribs, leaving most of the midriff intact. Supporting the body at the rear legs with our feet, we exert a firm pull to remove the internal organs, viscera, fat, and kidneys, placing them on plastic foil.

**Figure 1:**
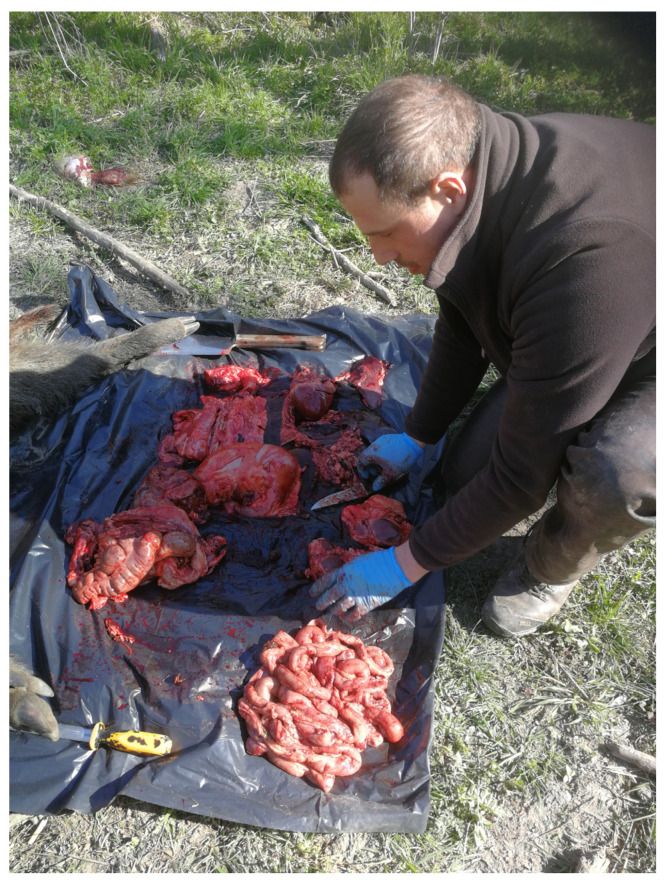
Only the organs we need are found in the middle of the spread foil (Original).

The internal organs and viscera are then separated, with the stomach and small intestine isolated from the large intestine at their junction. The connective tissue surrounding the duodenum and ileum is tightly adherent, making manual separation difficult. The small intestine is embedded in less dense connective tissue near the colon, facilitating easier separation. The connective tissue around the small intestine is removed from the foil. No extraneous organs should be on the foil at this phase (see Figure 1), as intestinal contents flowing during separation are necessary for accurate examination and must be collected.

Finally, the stomach and small intestine, freed from connective tissue, are placed at the centre of the foil. The foil is then folded and secured, and the contents are transferred to a prepared transport and storage container for further examination [26].

#### 3.1.2. Collecting Samples during Large-Scale Hunting

During large-scale hunts, our presence often serves dual purposes: conducting research and performing other essential tasks such as eviscerating and weighing game, preliminary examination of game meat, preparing kills, and conducting other diagnostic sampling. The intense work pace, especially during hunting breaks when game is brought in, necessitates additional assistance to carry out sampling and administrative tasks efficiently.

The method of removing viscera during large-scale hunts follows the same procedures described for individual hunts. However, to enhance efficiency, it is beneficial to cut through the cartilaginous symphysis of the sternum with a knife, eliminating the need to switch tools and allowing the entire operation to be performed in a single movement. This approach ensures sufficient time for separating the internal organs from the viscera.

For large-scale hunts, instead of using heavy-duty construction foil bags, we recommend using collection containers with a suitable capacity (15–20 L) that can be securely closed to prevent leakage. An 18–20 L plastic bucket with a lockable lid is ideal for this purpose. These buckets are cost-effective, easy to clean, and can be nested for efficient transport and storage. To facilitate sample identification, it is advisable to affix a wide strip of white insulating tape on the lids, which can be labelled and replaced as needed. These prepared collection containers should be lined up near the sampling area. Once a sample is placed in the container, it is closed with the lid, and the identification tag number is written on the lid with a permanent marker, ensuring well-isolated samples and precise data recording.

If time permits, removing the connective tissue bandage from the intestinal tract while it is still warm will facilitate subsequent examinations.

In cases where evisceration occurs in the field during the hunt, particularly in dense vegetation, it is practical to use a sample collection bag. Prepare foil bags according to the expected number of samples, affixing a strip of thick, white insulating tape for documentation purposes. These bags can be folded and carried in a backpack without adding significant weight. In the field, simply write the identification tag number on the bag, place the sample inside, and tie the bag securely with binding twine. Leave the bag next to the game for collection along with the wild animals.

### 3.2. The Second Phase of Sample Collection

#### Sampling in an Isolated Room under Controlled Conditions

For this procedure, it is essential to have a designated room or area equipped with running water (preferably both hot and cold) and a drainage system, ideally featuring an industrial floor drain or a large sink. This setup is crucial because the gastrointestinal sections, particularly the stomach, produce a substantial amount of fine particulate matter that necessitates removal with a strong, large jet of water. The drainage system must efficiently handle the large volume of water contaminated with sediment.

The examination of the intestinal tract and stomach is conducted separately. During evisceration, the faecal sludge extracted from the rectum is suitable for parasitological tests, as it remains uncontaminated by the environment. The examination process for the intestinal tract involves cutting sections from the entire small intestine, with an optimal length of 120 to 150 cm. This section is placed in a strainer, and a hose with a medium water jet is inserted into one end, filling the intestine section with water and washing its contents into the strainer. The hose should not exceed 0.5 inches in diameter to accommodate the smaller diameter of the intestines in subadults with a gutted body weight under 50 kg and to ease insertion in larger boars and sows.

Once the hose is inserted and secured by hand, approximately 15–20 cm from the intestine end, water flows through the section until clear water emerges from the opposite end, indicating thorough cleaning. The washed intestine section is then opened using one of several methods. A sharp knife can be used to cut the section from the inside out, exposing parasites attached to the intestinal wall.

**Figure 2:**
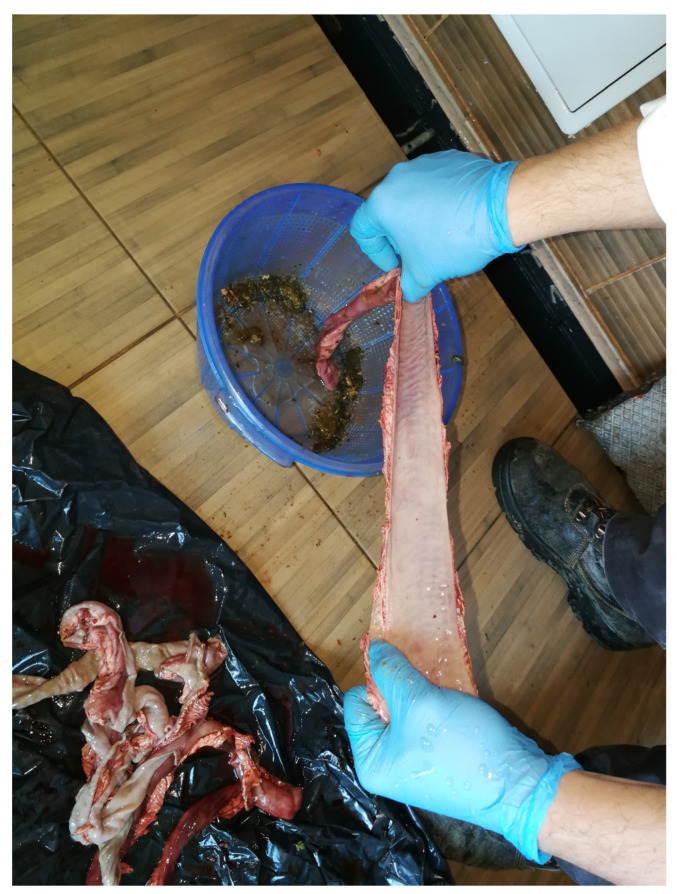
Our thumb is placed in one end of the washed intestinal section and the end of the intestinal section is continuously pulled, and the upper part of the intestinal wall is torn open at the tip of our finger (Original).

Our introduced method is more efficient, faster, and safer. It involves inserting a thumb into one end of the cleaned intestine section, pulling the section to tear the upper intestinal wall with the fingertip (See Figure 2). This technique spreads out the inner mucosa for clear visibility, allowing any remaining parasites and the injuries or deformities (bulla) they cause to be detected by touch. Specifically, the head of *M. hirudinaceus* attached to the intestinal wall creates a pea-sized, differently coloured diverticulum that can be easily felt and identified [27]. This method simultaneously opens and examines the intestinal section.

The intestinal contents collected in the strainer are thoroughly washed with a substantial volume of running water until only larger fibrous materials, endoparasites, and annelids consumed during feeding remain. Larger nematodes, such as roundworms, whipworms, and white-tailed worms, can be readily identified and distinguished by the naked eye when the intestinal sludge is passed through a strainer with a mesh diameter of 0.2–0.3 mm [28]. After allowing the contents to drain briefly, transfer them onto the examination table. Often, visible signs of infection can be observed during the washing process.

For the stomach examination, the stomach is first placed in the strainer. The dorsal side of the stomach is then incised from the oesophageal entrance to the intestinal exit, allowing the stomach contents to flow into the strainer. Rinse the remaining contents from the stomach into the strainer with ample running water. Next, spread the stomach wall upward, remove any mucus, and, if necessary, use a knife or scalpel to scrape and examine it thoroughly. Since the stomach contents frequently form a large mass that can block the fine mesh of the strainer, continue washing with water until only the larger fibrous materials and potential parasites remain and clean, sediment-free water passes through the strainer. After washing, drain the excess water and transfer the contents of the strainer onto a clean examination table. This table should be a 4–6 mm thick glass sheet measuring 80 cm × 120 cm, elevated at all four corners by approximately 15–20 cm, which allows for the placement of battery-powered or fixed lighting underneath the glass.

The inspection of the strainer contents follows the same procedure for both the stomach and the small intestine.

Prior to placing the sample on the table, prepare the necessary tools for isolating, preserving, and documenting the samples. These tools include a work table with a glass top, measuring 80 cm × 120 cm and illuminated from below; pre-labelled sealable containers for sample storage; a small or medium-sized, preferably dark-coloured bowl filled with clean water; a powerful, high-brightness headlamp; medical tweezers; and an alcohol-based marker.

**Figure 3:**
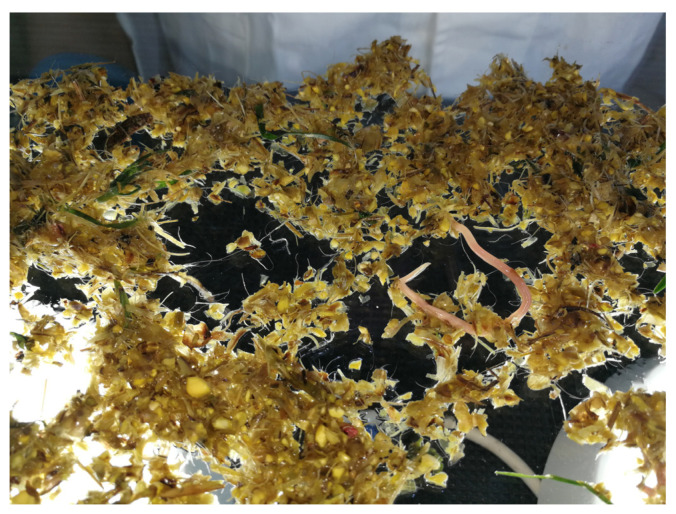
Spreading a sample containing *A. suum* on a glass table illuminated from below (Original).

Spread the sample evenly on the examination glass table to ensure it is sufficiently airy and transparent (see Figure 3). Rinse the sample in the prepared bowl of water to remove any adhering fibrous material, then transfer it to a sealable container or glass with an appropriate label. Once the strainer contents have been examined and deemed free of parasites, gather any residual fibrous material on the table to the centre and remove it using a rubber window scraper. This step prepares the table for the next sample.

The special big game identification number of the sample is written on the label of the container prepared for the storage of the sample, and the sample is placed in a refrigerator until the time of microscopic examination, stored at 4 °C.

## 4. Expected Results

A survey of faecal samples for parasite species identification and egg counting is less accurate than direct parasite counting during a dissection [29]. Compared to the methods employed by researchers [30], if there is an opportunity to collect samples from hunted game, it is advisable to use the dissection technique and examine the gastrointestinal tract for parasitological infection due to its superior accuracy. During our investigations, we successfully addressed several sampling complications by refining our methods, which were not initially apparent due to limited research experience. We outline the technical features and techniques that significantly streamlined and expedited our field sampling efforts by 30–60%. Precision and time efficiency are crucial for all researchers [31].

With practice, the cartilaginous junction located a few centimetres to the right or left of the sternum’s midline can be swiftly cut with a single knife stroke. This approach accelerates the process, which is particularly beneficial during large-scale hunts. The rectum can be drawn into the abdominal cavity through the pelvis, with the pelvic bone then cut using a saw, heavy knife, or splitting axe without risking damage to the intestinal tract. Cutting the pelvis before the rectum is fully retracted may lead to damage and subsequent sample loss. By cutting the sternum at the cartilaginous symphysis, the efficiency of our work is enhanced.

The internal organs and intestinal tract are removed together by holding the three-pronged wreath tape (lig. triangulare) securing the liver. By applying a pulling force in the direction of the pelvis while supporting the hind legs, the kidneys, surrounding fat, stomach, and entire intestinal tract can be extracted in one movement. These can then be placed on a prepared foil bag or covering foil. This method is suitable for initial sample collection for all visceral and partial muscle tissue endoparasitological examinations, as it provides access to all internal organs, the diaphragm, stomach, and complete intestinal tract for further dissection.

If the diaphragm separating the abdominal and thoracic cavities is cut, it becomes difficult to remove the thoracic organs, intestinal tract, kidneys, and associated fat simultaneously. Therefore, these organs should be removed together. In the case of kidney fat index tests, removing the kidney, surrounding fat, and abdominal cavity fat from a carcass lying on its back can be challenging. This method avoids tearing and ensures the complete removal of these components. Once removed, the kidney, along with the fat, can be easily rinsed and examined.

During individual sample collection or large-scale hunts involving multiple wild boars, it is advisable to remove the omentum from the stomach and small intestine while the organs are still warm. This practice simplifies subsequent work. When warm, the omentum is easily detachable; however, if it forms a ring on the intestinal wall, cutting the connective tissue with a sharp knife is necessary to prevent blockage. Removing the omentum from a cooled carcass can be difficult, resulting in a strip of connective tissue remaining on the intestinal wall. This strip can obstruct water flow during washing, increasing the risk of sample loss and rupturing the intestinal wall. To prevent this, ensure the lower end of the intestine hangs in the strainer or on a plastic foil to avoid sample loss.

For washing, use a rubber hose with a diameter of 0.25 inches, as larger hoses do not fit smaller intestines. If the hose diameter exceeds the intestinal diameter, insert the hose at least 5 cm into the intestine and hold it in place to facilitate effective washing. Raise the intestinal section slightly to ensure that the expelled contents are captured by the strainer, and increase the water flow and pressure until the intestine expands and water flows freely without contamination.

Cutting the entire length of the intestine with a knife or scalpel, as commonly performed by parasitological researchers [32], can be challenging due to potential snagging or deviation. This can complicate and slow down the process, particularly with smaller specimens. An alternative technique involves opening the intestinal segment with the thumb, which facilitates easier detection of parasites, whether free or embedded in the intestinal walls [32] and reduces the risk of damaging the sample.

During the washing process, strainers can become clogged, functioning as closed containers, which causes water to overflow and potentially wash away parasites. Instead of using a fixed 100-mesh standard sieve as used by other studies [33], it is more practical to employ a strainer with varying hole sizes—ranging from 50 to 70 mesh size, with smaller holes at the bottom and larger ones at the top. This design prevents clogging and maintains water flow.

Proper lighting from both below and above enhances the detection of parasites on the examination table. This setup allows for the easy removal of even the smallest parasites.

In large-scale hunting operations, having a dedicated administrator is essential. This role helps manage the workflow, as it is impractical to change rubber gloves frequently due to their tendency to tear and become cumbersome. Documentation should be performed with clean hands to avoid contaminating data sheets.

We hope this article provides a comprehensive overview of field sampling methods. The techniques described facilitate and simplify endoparasitological examinations of wild animals in field conditions, addressing the challenges outlined in our protocol and improving overall efficiency.

## Figures and Tables

**Table 1:** Wild boar endoparasitological examination methods.

Area	Reference	Year of Publication	Methods	Number of Examined Wild Boars
Turkey	[17]	2011	wild boars were examined by dissection	27 wild boars
West Spain	[20]	2013	wild boars were examined by dissection	300 wild boars
Southwest Iran	[18]	2016	Pathological and epidemiological studies were carried out	25 wild boars
Northwest Tunisia	[21]	2019	Dissection and faeces examination	591 wild boars
Denmark	[22]	2020	Dissection and faeces examination	255 wild boars
Poland	[14]	2020	Post-mortem examination	57 wild boars
Hungary	[23]	2024	Dissection	173 wild boars

Sampling methods of previous similar research.

## Data Availability

Data are contained within the article.
